# Management of Rheumatoid Arthritis: Possibilities and Challenges of Mesenchymal Stromal/Stem Cell-Based Therapies

**DOI:** 10.3390/cells12141905

**Published:** 2023-07-21

**Authors:** Yusuke Shimizu, Edward Hosea Ntege, Chinatsu Azuma, Fuminari Uehara, Takashi Toma, Kotaro Higa, Hiroki Yabiku, Naoki Matsuura, Yoshikazu Inoue, Hiroshi Sunami

**Affiliations:** 1 Department of Plastic and Reconstructive Surgery, Graduate School of Medicine, University of the Ryukyus, Nishihara 903-0215, Japan; 2 Department of Orthopedic Surgery, Graduate School of Medicine, University of the Ryukyus, Nishihara 903-0215, Japan; 3 Department of Plastic and Reconstructive Surgery, School of Medicine, Fujita Health University, Toyoake 470-1192, Japan; 4 Center for Advanced Medical Research, School of Medicine, University of the Ryukyus, Nishihara 903-0215, Japan

**Keywords:** mesenchymal stem cell, rheumatoid arthritis, autoimmune rheumatic disease, immunomodulation, cell therapy

## Abstract

Rheumatoid arthritis (RA) is a highly prevalent, chronic, and progressive autoimmune disorder primarily affecting joints and muscles. The associated inflammation, pain, and motor restriction negatively impact patient quality of life (QOL) and can even contribute to premature mortality. Further, conventional treatments such as antiinflammatory drugs are only symptomatic. Substantial progress has been made on elucidating the etiopathology of overt RA, in particular the contributions of innate and adaptive immune system dysfunction to chronic inflammation. Although the precise mechanisms underlying onset and progression remain elusive, the discovery of new drug targets, early diagnosis, and new targeted treatments have greatly improved the prognosis and QOL of patients with RA. However, a sizable proportion of patients develop severe adverse effects, exhibit poor responses, or cannot tolerate long-term use of these drugs, necessitating more effective and safer therapeutic alternatives. Mounting preclinical and clinical evidence suggests that the transplantation of multipotent adult stem cells such as mesenchymal stromal/stem cells is a safe and effective treatment strategy for controlling chronic inflammation and promoting tissue regeneration in patients with intractable diseases, including RA. This review describes the current status of MSC-based therapies for RA as well as the opportunities and challenges to broader clinical application.

## 1. Introduction

Rheumatoid arthritis (RA) is a chronic systemic autoimmune disease typically characterized by synovial inflammation, cartilage and bone destruction, and progressive joint deformities. It differs from osteoarthritis in cause, course, symptom profile, typical body location, and clinical management [1,2,3]. Patients with progressive RA are more likely to die prematurely (before the age of 75), primarily from cardiovascular and pulmonary diseases. In the early stages, the disease mainly affects the synovial joints, but eventually spreads to the lungs, blood vessels, and other parts of the body. Moreover, RA is among the most common autoimmune disorders, with an estimated global prevalence of 0.5–1.0% in 2002. Women are affected more often than men (4:1), and the incidence peaks between 40 and 60 years of age [1,4]. Therefore, RA severely reduces long-term quality of life (QOL), especially among women, and requires prolonged clinical management.

**Figure 1:**
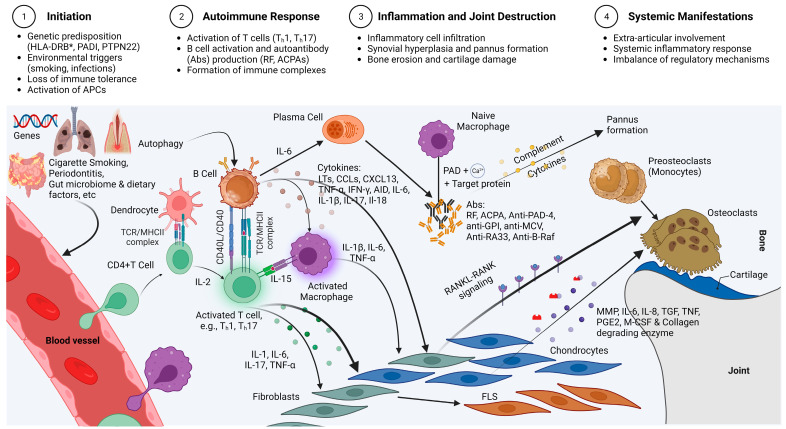
Scheme of the immunopathogenesis of RA, highlighting the intricate interplay of various cellular and molecular factors that play a pivotal role in RA development. The genetic background, including variants of human leukocyte antigen-DRB* (HLA-DRB*), peptidyl arginine deiminase (PADI) and protein tyrosine phosphatase non-receptor type 22 (PTPN22) genes, along with factors such as smoking, infections, and autophagy, contribute to the conversion of arginine to citrulline mediated by the peptidyl arginine deiminase (PAD) enzyme in the presence of calcium ions. The citrullination process affects target proteins such as fibrinogen, alpha-enolase, vimentin, and collagen type II. Antigen presentation by antigen-presenting cells (APCs), including dendritic cells, activate B cells and T cells through the CD40/CD40L interaction and the T-cell receptor/MHCII (TCR/MHCII) complex, respectively. B cells and T cells engage in a reciprocal activation loop, where B cells act as APCs for T cells, providing co-stimulatory signals via CD40/CD40L interaction, and T cells stimulate B cells through the TCR/MHCII complex. Th1 cells promote macrophage activation and secrete the pro-inflammatory cytokine tumor necrosis factor-alpha (TNF-α), while Th17 cells produce interleukin-17 (IL-17), interleukin-1 (IL-1), and TNF-α, influencing the function of chondrocytes, osteoclasts, and fibroblasts. The interaction between receptor activator of nuclear factor kappa-B ligand (RANKL) and receptor activator of nuclear factor kappa-B (RANK) also contributes to bone erosion by activating preosteoblasts. Autoantibodies, including rheumatoid factor (RF), anti-citrullinated protein antibodies (ACPA), anti-peptidyl arginine deiminase-4 (anti-PAD-4), anti-glucose-6-phosphate isomerase (anti-GPI), anti-mutated citrullinated vimentin (anti-MCV), anti-heterogeneous nuclear ribonucleoprotein A2/B1 (anti-RA33), and anti-B-Raf, form immune complexes and contribute to the formation of pannus. Key molecules such as activation-induced cytidine deaminase (AID), TNF-α, ILs, C-X-C motif chemokine ligand 13 (CXCL13), chemokines (CCLs), leukotrienes (LTs), interferon-gamma (IFN-γ), metalloproteinases (MMPs), prostaglandin E2 (PGE2), and collagen degradation enzymes are cytokines and enzymes produced upon activation of B and T cells. These molecules act in a coordinated manner to activate macrophages, fibroblasts, chondrocytes, and osteoblasts. During activation, fibroblasts transform into fibroblast-like synoviocytes (FLS), further contributing to the complex network of cellular and molecular interactions. Created with Biorender.com.

There is growing evidence that the etiopathology of conspicuous RA involves chronic inflammation resulting from innate and adaptive immune system dysfunction, including immune responses to autoantigens, abnormalities in the cytokine signaling network, and complement activation by immune complexes [5,6]. In contrast, recent reports suggest that the pathological processes underlying other phases of the disease, such as the early and refractory phases, may not be solely due to immune dysfunction. Rather, early RA is likely triggered by anti-citrullinated protein antibodies (ACPAs) via the interleukin (IL)/T helper cell (T_h_) cytokine pathway, while refractory RA is more likely due to cell-autonomous genetic and epigenetic perturbations involving transformed cell death pathways in synoviocytes following chronic inflammation [7] (See Figure 1).

Overt or clinically conspicuous RA is classified into two main subtypes depending on seropositivity to ACPAs [2]. About 67% of RA patients are seropositive for ACPAs, and ACPA-positive RA is a comparatively more aggressive disease. Hence, ACPAs are valuable diagnostic and prognostic markers for early or undifferentiated arthritis, exhibiting a high specificity of over 97% in clinical applications. In contrast, ACPA-negative RA has distinct genetic features and immune cell responses. Nonetheless, multiple genetic and environment factors influence the risk of both RA subtypes [1]. However, the precise pathological mechanisms underlying RA, especially the reasons for joint targeting in the early stages, remain elusive. Immune system dysfunction in RA is evidenced by high levels of glycosylated ACPAs in the blood circulation [7], the abnormal proliferation of FLSs [8], an overabundance of CD4+ cells (particularly T_h_17 cells) overexpressing the second isoform of the transcription factor RAR-related orphan receptor gamma (RORγt), reduced levels of suppressor T cells (T_reg_) expressing the transcription factor forkhead box protein P3 (FOXP3) (leading to an abnormal circulating T_h_17/T_reg_ ratio) [9], the presence of pathogenic B lymphocytes [10], an M1 (“proinflammatory”) to M2 (“immunomodulatory”) macrophage ratio > 1 [11], elevated levels of various proinflammatory cytokines, chemokines, and autoantibodies [12], and elevated oxidative stress markers [13]. Moreover, patients with RA may harbor distinct populations of extracellular vesicles (EVs) originating from the general circulation and joint synovial tissues, including EV exosomes containing microRNAs (miRNAs) implicated in RA pathogenesis or recovery such as miR-17 and miR-424 [14]. In addition, patients with RA may demonstrate the poor regulation of osteoblastogenesis and osteoclastogenesis in bone homeostasis [15,16,17].

In patients with progressive RA, ACPAs are less sialylated and galactosylated compared to patients at disease onset [7]. The hyperplastic synovium observed in RA may result from the hyperproliferation of FLSs, which in turn stimulate the expression and release of inflammatory cytokines such as TNF-α, IL-1β, IL-6, CXCL8, IL-12, IL-17A, IL-21, IL-22, IL-23, interferon (IFN)-α, and IFN-γ, as well as apoptosis resistance due to defects in tumor protein p53. Collectively, these responses result in further inflammatory joint damage and chondrocyte deficiency, leading to cartilage degeneration and the narrowing of the joint space [1]. Moreover, the abnormal T_h_17/T_reg_ and M1/M2 ratios lead to sustained overproduction of inflammatory cytokines that further damage joint tissues. In particular, TNF-α and IL-17 are considered vital to RA pathogenesis, as these proinflammatory factors trigger the release of degradative enzymes that proactively destroy cartilage matrix such as cathepsin K, MMP-1 and MMP-9, as well as additional downstream proinflammatory factors such as PGE2. Moreover, excessive TNF-α and IL-17 impair bone remodeling by enhancing the expression of osteoclastogenesis-mediating factors such as RANKL, macrophage colony-stimulating factor (M-CSF), and Semaphorin 4D (SE-MA4D). While a detailed description of bone remodeling is beyond the scope of this review, it is important to emphasize that immune dysfunction disrupts the normal balance between the formation and resorption of bone by osteoblasts and osteoclasts, respectively, ultimately promoting excessive bone absorption. The normal regulation of osteoblastogenesis and osteoclastogenesis requires the bidirectional transduction of activation signals through ephrin-B2-Ephrin type-B receptor 4, Fas ligand (FasL or Cluster differentiation (CD) 95L), and Semaphorin 3A-neuropilin-1 pathways. However, the cytokine stimulation of osteoblasts alters the expression and (or) secretion of multiple factors influencing osteoclast development, such as M-CSF, RANKL and its decoy receptor osteoprotegerin (thereby inhibiting RANK/RANKL signaling), fibronectin leucine-rich repeat transmembrane protein (FLRT2), netrin 1 and its receptor UNC-5 (participants in the FLRT2/netrin 1/UNC-5/RANKL axis), and the non-canonical WNT ligands WNT5A and WNT16. Moreover, osteoclasts influence osteoblast formation and differentiation by secreting soluble factors such as sphingosine-1-phosphate, SEMA4D, collagen triple helix repeat containing 1, component 3 (C3), and others [16,18]. All of these pathway present possible therapeutic targets for RA.

Circulating and synovial EVs originating from multiple cells, such as platelets, red blood cells, monocytes, lymphocytes, and other stromal cells, may contribute to RA pathogenesis by promoting T_h_ cell production, resulting in a shift in the T_h_17/T_reg_ ratio and ensuing overabundance of proinflammatory factors. For example, miR-17 in synovium-derived exosomes was reported to suppress transforming growth factor beta (TGF-β) receptor expression, and subsequently inhibit T_reg_ differentiation [14]. In addition, FLS-derived exosomes contain high levels of miR-424 under conditions such as low oxygen tension, and miR-424 is known to negatively regulate FOXP3 secretion and increase production of the inflammatory cytokines IL-17, IL-22, IL-1β, and TNF-α in RA model mice [19]. These molecules, particularly TNF-α, have also been reported to influence T cell-mediated cell death and sensitivity to apoptosis [20], and to promote immune complex formation in RA [21].

Numerous therapies have been developed based on an improved understanding of RA pathogenesis [22], including nonsteroidal antiinflammatory drugs (NSAIDs), corticosteroids, and disease-modifying antirheumatic drugs (DMARDs) such as conventional synthetic (c)DMARDs, targeted synthetic (t)DMARDs, and biological (b)DMARDs, also known as “targeted biologics”, “biologic agents”, or “biologics.” Among these agents, NSAIDs are the most frequently prescribed, particularly for the control of RA-related pain, while corticosteroids are also used for their strong anti-inflammatory properties. The cDMARDs, including methotrexate (MTX), leflunomide, and sulfasalazine (salazopyrin and salicylazosulfapyridine), reverse RA-related immune abnormalities and inflammation and are currently the first-line agents in rheumatologic therapy. The bDMARDs include antibodies that reduce inflammation by binding to and inhibiting cytokines and membrane-bound CD80/86 molecules on T cell-activated antigen-presenting cells (APCs). Further, bDMARDs treat not only inflammatory symptoms but also prevent joint deformation by suppressing bone and cartilage destruction and remodeling [22]. 

The prognosis for patients with autoimmune rheumatic disorders, including RA, has significantly improved in recent years. This improvement can be attributed to various factors, such as the increased use of bDMARDs and tDMARDs, both individually and in combination with cDMARDs. The identification of novel drug targets, including cell surface molecules, cytokines, epigenetic regulators, and signaling pathway components, has also contributed to this progress. Furthermore, the current clinical practice of targeted treatment, commonly known as “treat to target” (T2T), along with the close monitoring of disease activity, has played a crucial role [23].

Among the most promising recently identified drug targets are FLSs, pathogenic B lymphocytes, TNF-α, IL-1, IL-6, other cytokines, members of the Janus kinase (JAK) family, interleukin-1 receptor-associated kinase (IRAK) family members (particularly IRAK4), and various molecules involved in epigenetic regulation, such as DNA methylases, histone modifiers, and noncoding RNAs. New methods for disease monitoring, endorsed by organizations like the American College of Rheumatology (ACR), the European League Against Rheumatism (EULAR), and the Asia Pacific League of Associations for Rheumatology, have proven valuable for T2T clinical trials. These methods include composite scales like the joint Disease Activity Score based on C-reactive protein or Erythrocyte sedimentation rate (DAS-CRP/ESR), the Simplified Disease Activity Assessment Index, Health Assessment Questionnaire (HAQ), Western Ontario and McMaster Universities Osteoarthritis Index (WOMAC) and the Clinical Disease Assessment Index (CDAI) [24,25]. T2T has been found to be crucial in achieving remission or low disease activity (LDA) in patients with early or established RA [25].

A study by Hetland et al. [26] found that active conventional treatment with methotrexate combined with corticosteroids, particularly abatacept, was effective and safe for treatment-naive early RA. This study, along with a systematic review informing the 2019 update of EULAR recommendations by Kerschbaumer et al. [27], confirmed the efficacy of combining certain drugs with glucocorticoids for RA. TNF inhibitors, IL-6 receptor inhibitors, rituximab, and biosimilar DMARDs also showed effectiveness. Switching between bDMARDs demonstrated long-term safety and efficacy, and JAK inhibitors were also effective. Trials comparing different DMARD classes revealed similar response rates. Treatment targeting clinical remission was sufficient, while imaging remission led to more adverse events and higher costs. Tapering doses of certain drugs were feasible, and patients who experienced flares could regain their previous response.

However, patients aged between 40 and 60 require prolonged treatment to maintain remission in RA, which increases the risk of adverse effects and drug resistance [28,29]. Approximately 30% of RA patients may not respond to treatment or experience severe side effects, including bone marrow suppression, blood, liver, and/or kidney dysfunction, and infection [28,29]. Moreover, bDMARDs are contraindicated for RA patients with compromised immunity due to the heightened risk of opportunistic infections [28,29]. JAK inhibitors like Tofacitinib and Baricitinib are commonly used for treating RA [30]. Tofacitinib usage is associated with increased susceptibility to infections, and side effects such as headaches, hypertension, nausea, and elevated cholesterol levels [30,31,32]. Baricitinib can also increase cholesterol levels, and may cause respiratory tract infections and neutropenia [30,33,34]. Before starting JAK inhibitor treatment for RA, it is recommended to screen for infections and evaluate blood cell counts, as well as liver and kidney function [30,33,34]. JAK inhibitors should be avoided in certain conditions and during pregnancy, and caution should be exercised when combining them with other medications [30,33,34]. Perioperative management involves pausing JAK inhibitor therapy until proper wound healing is achieved [28]. Only a few ongoing developments, including selective JAK-1 inhibitors like Upadacitinib and Filgotinib, are being pursued [30,35,36].

Considering the challenges and potential limitations associated with current treatments, some RA patients may choose not to undergo long-term treatment or exhibit poor compliance. Therefore, it is crucial to develop alternative treatment methods that offer improved efficacy and tolerability [37,38]. In light of these considerations, further research into novel therapeutic approaches, including the utilization of mesenchymal stromal/stem cells (MSCs)-based treatments, is warranted. These innovative strategies aim to address the limitations of current therapies and leverage the regenerative and immunomodulatory properties of MSCs, ultimately enhancing the quality of life and long-term outcomes for RA patients.

## 2. The Ability of MSCs to Restore Health

Pluripotent stem cell-based therapies using embryonic stem cells (ESCs) and induced pluripotent stem cells (iPSCs) have demonstrated great promise for the treatment of refractory diseases in preclinical models and some clinical trials [39]. In addition, immune cell therapies, such as the adoptive transfer of regulatory T cells and chimeric antigen receptor T cell therapy, have been developed for the treatment of chronic inflammatory diseases [40,41]. In the following sections, we describe some of these therapies and the remaining challenges to routine clinical application.

Mesenchymal stem cells are a multipotent adult or somatic stem cell population that has long been used in preclinical studies, and more recently various MSCs have shown promise in clinical trials [39,42]. MSCs are adherent cells that are typically CD105+, CD73+, or CD90+, but rarely if ever CD45+, CD34+, or HLA-DR+ [39]. While MSCs may not be generally superior to iPSCs for accessibility, expandability, or pluripotency, they do, similarly to iPSCs, obviate ethical concerns associated with harvesting human ESCs, and can be obtained from the patient for personalized treatment [39]. Further, these cells can differentiate into multiple cell types of mesodermal origin, including osteocytes, chondrocytes, cardiomyocytes, and adipocytes, and so may be an equally feasible alternative to iPSCs for certain bone and joint diseases such as RA. In addition, MSCs have other desirable properties for clinical application, including low immunogenicity and strong immunomodulation capacity, as well as anti-inflammatory, proangiogenic, and anti-apoptotic activities. MSCs also produce EVs containing various trophic molecules promoting tissue repair or regeneration [39,42,43,44,45]. Through cell-to-cell contact and/or paracrine activity, MSCs release bioactive molecules that suppress the critical cytotoxic activities of large granular lymphocytes (natural killer or NK cells) and the development of APCs (dendritic cells or DCs), thereby inhibiting innate immunity. Also, MSCs have been reported to suppress the proliferation and activities of peripheral T_h_ cells, and induce T_reg_ formation and the release of factors such as indoleamine 2,3-dioxygenase (IDO), PGE2, TGF-β, histocompatibility antigen (HLA)-G5, and IL-10 to alleviate inflammation [39,46]. Theoretically, this low immunogenicity reduces the risks of MSC treatment, although the production of sufficient numbers for clinical application requires various isolation, culturing, and maintenance procedures that may carry certain risks, including the induction of inflammatory responses. In addition, MSC therapy is cost-effective [39,46], as these cells can be derived from numerous tissues depending on the end application. As MSCs from different sources have distinct differentiation potential spectra, specific tissues are preferred for RA treatment (Table 1) [45,46,47,48,49,50,51,52,53,54,55,56,57,58,59,60,61,62,63,64,65,66,67,68,69,70,71,72,73,74,75,76,77,78,79,80].

The immunomodulatory and anti-inflammatory capacities of MSCs are of primary importance for the effective management of autoimmune and chronic inflammatory diseases. However, cell-free MSC products may retain these properties with even greater safety. Thus, various procedures have also been developed to produce pure cell-free therapeutic products for RA such as MSC-derived exosomes (MSC-Exos) [81]. For instance, BM-MSC-derived TEMCELL^®^ HS Inj. (JCR Pharmaceuticals, Kasuga-cho, Japan) has been approved as the first allogeneic regenerative medicine in Japan for the treatment of acute graft-versus-host disease [82]. The mechanisms underlying the immunomodulatory and anti-inflammatory effects of MSCs and vesicular derivatives involved in controlling RA have been gradually unraveled [83,84,85,86]. This review summarizes current perspectives and evaluates the potential of MSC-based therapies for RA.

### 2.1. MSC-Based Therapies for RA

This review is based primarily on a wide literature survey of original English-language articles on regenerative medicine and RA treatment approaches. However, non-English articles were also evaluated for pertinence. The databases Medline, Embase, and Web of Science were searched using various combinations of the terms “stem cell,” “rheumatoid arthritis”, “autoimmune rheumatic disorder”, “autoimmune rheumatic disease”, “cell therapy”, “MSC immunomodulation”, “MSC immunosuppression”, “MSC antiinflammation”, “mesenchymal stem cells” and “immunomodulatory agents”. A web search was also conducted between July 2022 and October 2022 to identify clinical trials on cell therapies for RA and related diseases. In addition, the internet was searched for information on related clinical trials registered at ClinicalTrials.gov and domestic registries of Japan, including the Pharmaceuticals and Medical Devices Agency, Japan Pharmaceutical Information Center, Japan Registry of Clinical Trials, and Regenerative Medicine Provision Plans of the Ministry of Health, Labour and Welfare (MHLW) using search words like “rheumatoid arthritis”, “cell therapy”, “regenerative medicine products” and “rheumatism”.

#### 2.1.1. Mechanisms Underlying the Effectiveness of MSC-Based Treatments against RA

The healing properties of MSCs are directly affected by the level of inflammation in the tissue microenvironment (Table 2), suggesting that immune cell function is the primary therapeutic target of MSCs. Potential therapeutic mechanisms include paracrine activities of EVs and other soluble factors, as well as direct modulation through cell–cell contact [87,88,89,90,91].

A recent review by Bačenková et al. [92] highlighted several mechanisms influencing the efficacy of MSCs for modulating immune system function in RA. The conversion of the amino acid tryptophan to kynurenine in IDO influences the ability of this enzyme to impair the synthesis of several cellular proteins that suppress T_h_ cell proliferation, influence the formation of T_reg_ cells, and (or) induce tolerogenic dendritic cells (tDCs) [93]. The secretion of TGF-β by MSCs enhances T_reg_ development and M1/M2 stability, and influences monocyte differentiation toward tDCs [94]. In addition, MSCs reduce the ability of pathogenic B cells to migrate by secreting IL-10, TGF-β, PGE2, NO, and IDO. These factors also impact cell expansion and development, immunoglobulin (Ig) production, and C-X-C chemokine receptor 4 (CXCR4), CXCR5, and C-C chemokine receptor type 7 (CCR7) expression. In vitro studies have found that MSCs increase IL-10 production and secretion in the presence of CD3+ T cell populations. The CD3 protein complex acts as a T cell co-receptor involved in activating both cytotoxic T cells (from CD8+ naive T cells) and T_h_ cells (from CD4+ naive cells). Increased IL-10 levels enhance the capacity of MSCs to promote the expansion of T_reg_ cells and enhance programmed cell death protein 1 (PD-1) expression by immunomodulation-associated CD4+ and CD25+ cells (naturally occurring T_reg_) [87]. MSCs also express several molecules with immunomodulatory functions, such as HLA-G1, which binds the Ig-like transcript 2 receptor to inhibit IFN-γ secretion, B cell proliferation and differentiation, antibody secretion, and T cell chemokine expression. Programmed cell death protein 1 (PD-1) ligand expression by MSCs suppresses T_h_ cell production in several diseases, including autoimmune diseases, while the APC type I transmembrane protein CD40 is essential for Th cell activation. Also, MSC-expressed Jagged-1 interacts with the Notch signaling pathway to initiate a cascade of proteolytic cleavages and activate the transcription of downstream target genes encoding T_h_ cell-inhibitory proteins. Activated MSCs express adhesion molecules, such as vascular cell adhesion molecule 1 (VCAM-1 or CD106), intracellular adhesion molecule 1 (ICAM-1/CD54), and CXCR4, which promote cell homing by binding to various extracellular matrix (ECM) molecules, resulting in enhanced MSC migration and interaction with immune cells. Indeed, ICAM-1 and VCAM-1, which are induced by IFN-γ and IL-1, have substantial effects on MSC-mediated immunomodulation [95]. MSC-derived chemokines affect lymphocytes that migrate to the sites of inflammation and also bind to CCR5 and CXCR3 expressed on T cells. Activated MSCs have a greater capacity to suppress NK cell activities at the site of inflammation. Furthermore, MSCs express CD90, the activated leukocyte adhesion molecule CD166, and other integrins that mediate interactions with T cells. MSC regulated inflammation by suppressing T_h_1 and T_h_17 proliferation as well as by expansion of T_reg_ cells. Lefevre et al. [8] recently demonstrated the ability of MSCs to modulate the immune system by reducing the harmful T_h_1/T_h_17 response and enhancing protective T_reg_ cell responses. Najar et al. [96] also observed the inhibition of lymphocyte proliferation during the co-cultivation of MSCs and mitomycin agglutinin-activated T cells. In summary, T_h_17/T_reg_ and M1/M2 imbalances are seminal to RA immunopathogenesis, and MSCs demonstrate the capacity to modulate the immune system by influencing the activities of memory T lymphocytes, including T_h_17 cells, and by promoting T_reg_ cell expansion [97,98].

#### 2.1.2. Merits of MSCs for RA Therapy

The use of MSCs for cell-based RA treatment is garnering intense interest owing to the documented capacity of these cells to regulate a wide range of basic cellular functions, and the low inherent immunogenicity resulting from minimal expression of MHC class I and the absence of MHC class II and costimulatory molecules CD40, CD80, and CD86. Hence, the number of clinical studies has increased approximately four-fold over the last decade, although fewer than 10% are phase III or IV trials [92].

The administration of MSC-EVs can regulate immune function both locally and at distant sites. Further, EVs derived from MSCs of different tissue origins may be safer cell-free therapeutic alternatives for various diseases, including RA [59,99,100]. Indeed, MSC-derived EVs have demonstrated bioactivities similar to the source MSCs and are also capable of modulating MSC-mediated tissue regeneration [44,59,100,101]. Like EVs from other sources, MSC-derived EVs are categorized into exosomes (MSC-Exos) and ectosomes (MSC-Ectos). MSC-Exos range in size from 40 to 160 nm, and exhibit surface marker profiles similar to the parent MSCs in addition to distinctive CD63, CD81, and CD9 expression profiles [102], while ectosomes are more heterogeneous and include microvesicles (MVs) and microparticles ranging in size from ~50 nm to 1 μm. The cargo of a typical MSC-EV includes a large array of endosomal sorting complexes required for transport proteins I–III, such as tumor susceptibility gene 101 and heat shock protein 70, in addition to apoptosis-linked gene 2-interacting protein X, cytoskeletal proteins, various metabolites (such as deoxyribonucleic acids), mRNAs, and noncoding RNAs [99,103,104,105]. Like the source cells, MSC-Exos and MVs are proangiogenic antigen-presenting entities that suppress inflammation and regulate the immune system [106,107,108,109].

#### 2.1.3. MSC-Based Therapy: Preclinical RA Studies

##### MSC Administration

Numerous preclinical studies have demonstrated the therapeutic efficacy of MSCs in models such as collagen-induced arthritis (CIA) [110,111]. For instance, human MSC transplantation was reported to improve the LDA score in the CIA model [112,113]. The transplantation of human umbilical mesenchymal stem cells (hUC-MSCs) also induced the significant downregulation of proinflammatory cytokines and osteoclastogenesis, while enhancing inflammatory mediators in the injured ankle joints of mice [114]. Transplantation of MSCs improved paw edema, suppressed rheumatoid factor (RF) elevation, enhanced antioxidant capacity, and reduced NF-κB, TLR-2, MMP3, and cartilage oligomeric matrix protein-1 expression levels in a rat model of RA compared to hematopoietic stem cell transplantation or administration of the clinical antirheumatism drug methotrexate (MTX) [115]. A combination of transplanted MSCs and cytokines also effectively reduced synovitis and systemic inflammation in the CIA mouse model, as evidenced by substantial reductions in RF (64%), CRP (80%), anti-nuclear antibodies (ANAs, 71%), TNF-α (63%), and monocyte chemoattractant protein-1 (MCP-1, 68%) [116]. Nam et al. [112] reported that peritoneal lavage cells from mice treated with MSCs expressed higher levels of stromal cell-derived factor 1-alpha (SDF-1α or CXCL12) and RANTES than controls, again consistent with a possible therapeutic utility for RA as MSC migration was enhanced in the presence of SDF-1α and/or RANTES. MSCs also induced T_h_ cells to differentiate into T_regs_ in vitro, and the expression of FOXP3 mRNA was upregulated in the forepaws of MSC-treated CIA model mice [23]. The transplantation of allogeneic MSCs suppressed T follicular helper (Tfh) cell differentiation in RA patients in part by enhancing IDO production, suggesting that arthritis progression may be prevented by MSC transplantation [117]. A review by Roudsari et al. [118] also highlighted studies showing that MSCs regulate T cell apoptosis through the FasL/Fas pathway, induce immune tolerance, and improve joint function in the CIA mouse model. Further, BM-MSCs were reported to improve bone erosion in CIA model rodents by inhibiting factors associated with osteoclastogenesis and by promoting differentiation into chondrocytes [119].

##### MSC-Derived EVs 

Miao et al. [14] recently summarized several preclinical studies on MSC-EVs as cell-free therapy for RA in model animals. Cosenza et al. [44] were the first to demonstrate that MSC-Exos were more immunosuppressive than microparticles in CIA model mice. Zhange and colleagues recently reported that BM-MSC-Exos could delay the progression of osteoarthritis by alleviating cartilage damage, reducing osteophyte formation and synovial macrophage infiltration, inhibiting M1 macrophage production, and promoting M2 macrophage generation in osteoarthritis model rats [45]. Additionally, the expression levels of IL-1β, IL-6, and TNF-α in synovial fluid were reduced, and the release of IL-10 was increased. In vitro, macrophages treated with MSC-Exos maintained the chondrogenic characteristics of chondrocytes and inhibited hypertrophy [45,46]. 

A recent review also concluded that MSC-Exo-derived miRNAs have immense potential for future clinical applications [105]. A study of RA treatment by targeting FLAs with BMSC-EVs containing miR-34a reported notable efficacy via the cyclin I/ataxia-telangiectasia mutated symbol/ataxia-telangiectasia and Rad3-related protein/p53 signaling pathways [99]. Also, BM-MSCs-Exos containing miR-192-5p suppressed CIA progression in rats via ras-related C3 botulinum toxin substrate 2 [120], and BM-MSC-Exos containing miR-320a repressed RA-FLS proliferation by downregulating CXC chemokine 9 [121], while MSC-Exos containing miRNA-150-5p reduced angiogenesis and FLS proliferation in RA patients and CIA model mice by downregulating MMP14 and vascular endothelial growth factor [122]. Su et al. [123] recently demonstrated that MSC-Exos also participated in the intercellular transfer of the long noncoding RNA heart- and neural crest derivatives-expressed protein 2 antisense 1, and suppressed the activation of RA-FLSs via miR-143-3p/tumor necrosis factor and alpha-induced protein 3/NF-κB pathways in RA patient synovial tissue and cultured cells, suggesting the potential involvement of these MSC-Exos-evoked signaling cascades in RA pathogenesis and as possible treatment targets.

Recent studies have also examined using AD-MSC-EVs for RA treatment. One particularly promising study reported that AD-MSC-EV administration improved RA in the IL-1ra knockout mouse model [124]. The authors found that joint swelling was reduced to near control levels by AD-MSCs and separately by AD-MSC-EVs, and that these effects were associated with decreases in proinflammatory cytokine concentrations. These effects may be mediated by the suppression of T_h_ cells, as known T_h_ cell factors suppressing proinflammatory cytokine expression are also major constituents of AD-MSC-EVs [68,69,70]. However, the impact of AD-MSC-EVs is highly dependent on MSC type (autologous vs. allogeneic), handling processes, and site of tissue extraction [42,72,73,100,125,126,127,128,129]. 

MSC-EVs from umbilical cord have demonstrated superior efficacy against CIA compared to MSCs from other tissues, and even compared to antirheumatic drugs such as MTX [105]. However, the efficacy of UC-MSC-EVs is also highly dependent on MSC handling procedures and levels of the transcriptional factor FOXP3 [130,131,132,133]. In addition to these conventional sources, MSC-EVs isolated from olfactory epithelium have demonstrated immunosuppression through a reduction in T_h_ cell proliferation and increased T_reg_ cell production in vitro [101]. In CIA model mice, the adoptive transfer of olfactory epithelium-derived MSCs markedly delayed arthritis onset and decreased disease severity, and these clinical outcomes were accompanied by T_reg_ expansion and reduced T_h_1/T_h_17 cell responses in vivo [134]. Also, MSC-Exos obtained from gingival and other tissues have demonstrated immunosuppression by reducing angiogenesis and the M1/M2 polarization ratio, and by improving chondrogenesis and bone remodeling [135,136,137,138,139].

#### 2.1.4. Studies on MSC-Based Therapy for RA in Japan

In Japan, research on MSC-based cell therapies for RA remains very limited. Currently, there are no complete or ongoing MSC-based clinical investigations or trials for RA, and only a single MHLW-registered study by Trinity Clinic, Fukuoka, titled “Treatment of RA using Autologous Adipose-derived Mesenchymal Stem Cells”, was identified.

#### 2.1.5. Studies on MSC-Based Therapy for RA in Other Counties

Several clinical trials on MSC-based therapies against RA (Table 3) were identified by searching various trial registries and internet sites. Of these, UC-MSCs were the most common cell type examined, followed by AD-MSCs and BM-MSCs. The largest number of trials was conducted in the United States of America, followed by China, Korea, and the European Union. The remaining studies were conducted in Iran, Jordan, and Panama. Early phase trials (Phases I and II) accounted for more than 80% of the total.

In a recent review by Lopez-Santalla et al. [140], MSC-based therapy trials for RA were well summarized. Groundbreaking and completed studies in this field have demonstrated promising safety and efficacy, particularly in refractory patients, with long-term effects observed without significant adverse events [2,3]. These findings align with results from other immune-mediated diseases [141,142].

Among the highlighted studies, a pilot trial in Korea (2010) enrolled 10 patients with autoimmune diseases, including 4 with RA. Autologous AD-MSCs were expanded for multiple infusions, with doses ranging from 2 × 10^8^ to 3.5 × 10^8^ cells per patient. The trial observed clinical benefits, and up to 8 × 10^8^ cells were safely administered within a month [143].

In another trial in China (2012), four RA patients received allogeneic MSCs via intravenous (IV) infusion. Although remission was not achieved, three patients showed a moderate response for several months [144].

A randomized trial in Spain (NCT01663116) involved 53 refractory RA patients who received allogeneic expanded ADMSCs (eASCs) in different doses. Promising outcomes were observed after 6 months of monitoring. The IV infusion of Cx611 in RA patients was well-tolerated, without dose-related toxicity. Positive responses based on EULAR criteria, DAS28-ESR, and CRP were observed for up to three months compared to the placebo group. However, the refractory profile of the RA patients may have limited the beneficial effects of MSC therapy. The trial did not observe significant changes in T cell populations or adverse clinical consequences, but 19% of patients developed MSC-specific anti-HLA-I antibodies [145].

At the Hospital of People’s Liberation Army Air Force in China, 172 RA patients received a single IV dose of allogeneic UC-MSCs (NCT01547091). No serious adverse effects were reported, and significant remission was observed based on ACR criteria, DAS-28, ESR, HAQ, and decreased inflammatory markers. Beneficial effects were observed in the short-term (up to 8 months) and long-term (up to 3 years) compared to the control group [146,147].

At Daping Hospital in China, 53 refractory RA patients received a single IV dose of allogeneic UC-MSCs (ChiCTR-ONC-16008770). No serious acute adverse events were reported, and clinical safety and efficacy were confirmed. Approximately 54% of MSC-treated patients showed a good or moderate response, while 46% had no clinical response compared to the control group. Transient effects were observed, and additional MSC infusions were suggested for sustained effects. Serum IFN-γ levels were associated with clinical benefits, and could serve as a predictive biomarker [148].

A preclinical study emphasized the role of IFN-γ signaling in MSC immunomodulatory effects. Treatment with INF-γR^−/−^ MSCs had no therapeutic effects on RA progression, indicating the dependence of MSC efficacy on IFN-γ levels. The immunosuppressive properties of MSCs rely on an inflammatory microenvironment for optimal functioning [147].

In a 2017 clinical trial (ChiCTR-INR-17012462), Xu et al. [149] treated 63 refractory RA patients with UC-derived MSCs and recombinant IFN-γ. The combination significantly improved clinical efficacy from 53.3% to 93.3% without safety issues during the 1-year follow-up. 

In a phase I trial (NCT03333681), nine refractory RA patients received autologous BM-MSCs alongside conventional therapy. No complications or adverse events occurred, and significant improvements in disease activity measures were reported. Although certain serum cytokine levels did not show statistical differences, there was an increase in regulatory CD4+ T cells and a decrease in Th17 cells. B-cell response and proliferation decreased, and plasma levels of CXCL8, CXCL12, and CXCL13 were reduced for up to 1 year [51,52,150,151].

Kang Stem Biotech funded two trials (CURE-IV and FURESTEM-RA) in Korea. These trials involved treatment-naïve RA patients receiving allogeneic UCB-MSCs. The phase I trial (NCT02221258) showed no serious adverse events, improvements in disease activity measures, and reduced inflammatory cytokine levels. However, limitations included a small sample size and short follow-up. A 5-year observational study is ongoing, and a phase I/IIa randomized double-blind placebo-controlled trial (NCT03618784) is currently underway [152].

**Table 3:** Clinical studies of cell therapies against RA conducted worldwide.

Trial Registration/ Country	Phase	Allo/Auto	Tissue Source	Regimen	Enhancement Method	Enrollment	Start Year	Completed/ Reference
EudraCT: 2010-021602-37; NCT01663116/Spain	Ib/IIa	Allo.	Adipose tissue	IV weekly infusions of ADMSCs: 1 million/kg (cohort A), 2 million/kg (cohort B), 4 million/kg	None	53 active refractory RA patients	2011	Yes [145]
Unidentified/Korea	Pilot	Allo.	Adipose tissue	IV 2 doses of 3 × 10^8^/patient and 4 doses of 2 × 10^8^/patient and 1 dose of 2 × 10^8^/patient; +1 IA dose of 1 × 10^8^/patient or IV single dose of 3.5 × 10^8^/patient + 1 IA dose 1.5 × 10^8^/patient;	None	4 refractory RA	2011	Yes [143]
Unidentified/China	Pilot	Allo.	Bone Marrow/Umbical Cord	IV single dose of 1 × 10^6^ cells	None	4 refractory RA	2012	Yes [144]
NCT01547091/China	I/II	Allo.	Umbilical Cord	IV 4 × 10^7^ cells/patient	UC-MSC+DMARDS	172 patients with RA were recruited/64 patients monitored for three years	2013	Yes [146,147]
NCT01851070/USA	II	Allo.	Bone Marrow	IV single dose of 1 or 2 × 10^6^ mesenchymal precursor cells (MPCs)	None primed precursor cells	48 refractory RA	2013	Yes [140,153]
NCT01985464/Panama	I/II	Allo.	Umbilical Cord	Unknown	Unknown	20 refractory RA	2013	Unknown [extracted from ‘www.ClinicalTrials.gov’; accessed on 23 June 2023;]
NCT02221258/Korea	I	Allo.	Umbilical Cord Blood	IV single dose of 2.5 × 10^7^, 5 × 10^7^, or 1 × 10^8^ cells of hUCB-MSCs/patient	None	9 RA patients with moderate disease activity	2014	Yes [extracted from ‘www.ClinicalTrials.gov’; accessed on 23 June 2023;]
NCT02643823/China	I	Allo.	Umbilical Cord	IV weekly single dose of 2 × 10^7^ hUC-MSC for 1 month	hUC-MSC + DMARDs	40 refractory RA	2016	Unknown
ChiCTR-ONC-16008770/China	I	Allo.	Umbilical Cord	IV single dose of 1 × 10^6^ UC-MSCs/kg of body weight	None	53 refractory RA	2016	[148]
NCT03067870/Jordan	I	Auto.	Bone Marrow	Unknown	Unknown	100	2016	Unknown
NCT03333681/Iran	I	Auto.	Bone Marrow	IV single dose of 1 to 2 × 10^6^ cells/kg of body weight	I/V hydrocortison (100 mL) and oral dimenhydrinate (10 mL) before MSCs administration + conventional therapy	15 refractory RA	2016	Yes [extracted from ‘www.ClinicalTrials.gov’; accessed on 23 June 2023;]
ChiCTR-INR-17012462/China	I/II	Allo.	Umbilical Cord	IV single daose of 1 × 10^6^ cells	UC-MSCs combined with recombinant IFN-γ.	63 refractory patients	2017	Yes [149]
NCT03186417/USA	I	Allo.	Umbilical Cord	IV single dose of 2, 4 or 6 × 10^6^ MPCs	None	10 new onset RA actual enrolment	2017	Unknown [153]
NCT03798028/China	II/III	Allo.	Umbilical Cord	IV single dose of 1 × 10^6^ cells/kg of body weight of UC-MSCs	+conventional treatment	250 DMARD-resistant RA patients	2017	Unknown [extracted from ‘www.ClinicalTrials.gov’; accessed on 23 June 2023;]
NCT03618784/Korea	I/II	Allo.	Umbilical Cord Blood	IV, 5.0 or 10 × 10^7^/patient; 3 doses	FURESTEM-RA Inj.+DMARDs	33 refractory RA	2018	Unknown [extracted from ‘www.ClinicalTrials.gov’; accessed on 23 June 2023;]
NCT03691909/USA	I/IIa	Auto.	Adipose tissue	IV single dose of 2 × 10^8^ adMSCs	HB-adMSCs	15 with active RA	2018	Yes [extracted from ‘www.ClinicalTrials.gov’; accessed on 23 June 2023;]
NCT03828344/USA	I	Allo.	Umbilical Cord	IV single dose of BX-U001 at 0.75 × 10^6^ cells/kg of body weight (Cohort 1) or 1.5 × 10^6^ cells/kg of body weight (Cohort 2)	Unknown	16	2020	Not yet known [extracted from ‘www.ClinicalTrials.gov’; accessed on 23 June 2023;]
NCT02903212/France	I/II	Auto.	Peripheral blood leukocytes	Unknown	Rendered apoptotic by X-ray irradiation	22	2021	Not yet known [extracted from www.ClinicalTrials.gov; accessed on 23 June 2023;]
NCT04170426/USA	I/IIa	Auto.	Adipose tissue	IV three doses of 2.0–2.86 × 10^6^ cells/kg on day 1, 4 and 7	Celltex-AdMSCs with unknown enhancement strategy	54	2021	Not yet known [extracted from www.ClinicalTrials.gov; accessed on 23 June 2023;]
NCT04971980/China	I/II	Allo.	Umbilical Cord	IV single infusion of hUC-MSC at 0.5 × 10^6^ cells/kg, or 1.0 × 10^6^ cells/kg, or 1.5 × 10^6^ cells/kg in three cohorts	Unknown	9	2021	Not yet known [extracted from www.ClinicalTrials.gov; accessed on 23 June 2023;]
NCT05003934/USA	I	Allo.	Umbilical Cord	IV single infusion of 100 million cells AlloRx	Unknown	20	2022	Not yet known [extracted from www.ClinicalTrials.gov; accessed on 23 June 2023;]

Allo, Allogeneic; Auto, autologous; ChiCTR-INR, Chinese registry, “www.Chictr.org”; accessed on 23 June 2023; EudraCT, European Union Drug Regulating Authorities Clinical Trials Database; IA, intra-articular; IV, intravenous; MPCs, multipotent progenitor cells; NCT, The National Clinical Trial; UC, umbilical cord; USA, United States of America.

Mesoblast Ltd. conducted a phase II trial (NCT01851070) in the USA in 2013, using allogeneic MPCs derived from BM. The trial involved RA patients with incomplete responses to TNF-α inhibitors. The highest MPC dose showed significant treatment benefits, improving clinical symptoms and disease activity. Further assessment and dose optimization are needed. Another trial using MPC-based therapy for RA patients is underway in the USA (NCT03186417) [153].

In the report by Kabat et al. [154], the optimal protocol for MSC therapy in RA patients remains undefined. Allogeneic MSCs are commonly used (78%) due to challenges in obtaining autologous MSCs. MSCs derived from bone marrow, adipose tissue, and umbilical cord show comparable safety and efficacy. Most trials administer less than 10 × 10^6^ MSCs per kilogram in a single infusion. The correlation between dose and efficacy is inconclusive, although higher cell doses above 1 × 10^6^ cells per kilogram may be more effective. High MSC doses (8 × 10^8^ MSCs per patient) have been reported as safe. However, most trials have small sample sizes and lack placebo groups. Furthermore, enrolled patients often have longstanding refractory RA. It is worth noting that early treatment appears to be more effective in other inflammatory conditions [147].

Currently, several ongoing clinical trials for MSC-based therapy in RA are being conducted, each with different MHC backgrounds, tissue sources, and cell doses. The trials include both allogeneic and autologous approaches, with umbilical cord tissue being the most common source [146,147].

These ongoing trials include a study by Wang et al. (7) at the Stem Cell Institute in Panama (NCT01985464), which is using allogeneic UC-MSCs to treat 20 DMARD-resistant RA patients. The trial aims to evaluate adverse events, biological efficacy, and immunological parameters after one year [146,147].

Another trial (NCT03067870), sponsored by Stem Cells Arabia in Jordan, started in 2016, seeking to assess the safety and efficacy of autologous BM-MSCs in RA patients. The study plans to enroll 100 patients and monitor systemic efficacy using VAS scores for one month and WOMAC scores and imaging for six months to assess joint regeneration potential [146,147].

A multicenter trial (NCT03798028) at Xijing Hospital in China, initiated in January 2019, is recruiting adult RA patients with moderate or severe RA and anemia and/or interstitial lung disease. The trial aims to evaluate the safety and therapeutic efficacy of allogeneic UCB-MSCs over 24 weeks [146,147].

A proof-of-concept Phase I trial began in 2017 at MetroHealth Medical Center in Cleveland, OH, USA (NCT03186417). The study aims to recruit 20 newly diagnosed RA patients and assess the safety and efficacy of allogeneic BM-MSCs over 24 months using Patient-Reported Outcomes Measurement Information System Computer Adaptive Test (PROMIS CAT), Routine Assessment of Patient Index Data 3 (RAPID3) questionnaire, and DAS28-CRP [146,147].

In 2018, Kang Stem Biotech Co. Ltd. initiated a Phase I/IIa RA trial in Korea (NCT03618784) to evaluate the efficacy of intravenous allogeneic UC-MSCs in 33 refractory or intolerant RA patients. The study monitors patients for 16 weeks using various scoring systems and cytokine level analyses [146,147].

Hope Biosciences in TX, USA, has been conducting a phase I/II trial (NCT03691909) since 2018 to evaluate the safety and efficacy of autologous ADMSCs in RA patients. The trial is assessing multiple factors for up to 12 months [146,147].

Baylx in CA, USA, recently initiated a Phase I trial (NCT03828344) to evaluate the safety and effects of allogeneic UC-MSCs in refractory RA patients. The trial assesses multiple criteria and is expected to be completed in September 2022 [146,147].

Finally, Celltex Therapeutics Corporation in Houston, TX, USA, is currently enrolling patients in a Phase I/IIa trial (NCT04170426) to treat DMARD-refractory RA patients with autologous ADMSCs. The trial’s expected completion date is December 2023 [146,147].

#### 2.1.6. Challenges and Strategies for Enhancing MSC-Based Treatments for RA

MSCs represent a promising therapeutic option for various diseases, including RA. However, several challenges need to be addressed to optimize MSC-based therapies. These challenges arise from multiple factors, such as the tissue of origin, donor gender, age, health status, and/or medical history of the MSCs, as well as the processing of the tissue, culture conditions, freezing and thawing of the cells, and administration routes. These factors significantly influence the outcomes of MSC-based therapies and necessitate urgent optimization [23,66,155,156,157].

Firstly, the donor’s health condition can have an impact on various aspects of MSCs obtained, including their quantity, quality, regenerative potential, immunomodulatory capabilities, and secretome, as factors such as age, inflammatory/metabolic status, medications/treatments, and infectious diseases play a role in modifying MSC properties, thereby leading to significant heterogeneity in terms of surface markers, differentiation capacities, and physiological functions of MSCs [158]. The current minimal criteria for defining MSCs are insufficient to capture this heterogeneity, requiring further exploration to identify specific subsets and characterize their functional properties [158,159]. The migratory capacity of MSCs is crucial for their therapeutic efficacy [160]. However, the expression profiles of chemokines in damaged tissues often do not match the receptor profiles on MSCs, resulting in suboptimal migration rates. To enhance migration, genetic modifications of MSCs to express specific chemokine receptors have been explored, and the choice of delivery route also affects homing and paracrine functions [161,162].

Another challenge is the limited expansion capacity of MSCs, which makes it difficult to obtain a sufficient number of cells for clinical trials [161,163]. Prolonged culture duration and increased passage numbers lead to decreased proliferation, altered morphology, and compromised viability [157,164]. Therefore, optimizing culture conditions and developing scalable manufacturing processes are essential to overcome this limitation [165].

Safety concerns associated with specific tissue sources of MSCs, such as UCB-MSCs, also need to be addressed [61]. Chromosomal abnormalities and potential tumorigenicity have been reported, necessitating efforts to ensure the safety of MSC-based therapies. The cloning of single cells derived from UCB-MSCs and the use of specific antigens for cell isolation have been explored, but a universally accepted culture protocol is yet to be defined. Adherence to GMP is crucial, and the use of bioreactors and automated systems offer controlled environments and improved scalability for large-scale production [54,166,167]. Additionally, frozen preservation techniques are being developed to enhance long-term cell viability and facilitate storage and transportation [168].

To optimize MSC therapies, various strategies have been investigated. Biomaterial strategies aim to improve MSC function, but immune responses may be triggered [169]. Loading MSCs with small-molecule encapsulating microparticles or utilizing decellularized ECM scaffolds and synthetic polymers are alternative approaches [161]. Genetic modifications using viral or non-viral vectors have been explored to enhance therapeutic potential, but associated risks need to be carefully considered [161]. Human-induced pluripotent stem cell-derived MSCs and CRISPR/Cas9 technology show promise as well [161]. “Priming” MSCs and utilizing an MSC-derived secretome, including extracellular vesicles (EVs), have been effective in enhancing therapeutic function [170]. MSC-derived EVs have shown promise in clinical trials, and the fields of artificial intelligence (AI) and engineered EVs offer exciting prospects for advancing MSC therapy [161]. AI can accelerate drug development and improve understanding of MSC therapies, while engineered MSC-EVs can be modified to enhance therapeutic potential [161].

In the context of RA, several strategies have been explored to enhance the therapeutic effects of MSCs [156,157,158,159,160,161,162]. These strategies include coculture methods, growth factors, cytokines, receptor agonists, hypoxia, autophagy, and genetic modification [23,156]. Summarized in the report by Sarsenova et al. [156], combining MSCs with IL-10-producing T_reg_ cells has shown enhanced effectiveness in suppressing inflammatory responses and preventing destructive arthritis in mice. Culturing MSCs as 3D spheroids has also been proposed to enhance their immunomodulatory and anti-inflammatory properties through increased TSG-6 and COX-2 expression. Targeting immune receptor agonists, such as TLR3 and TLR4, has shown improved cellular properties and immunomodulation. Preconditioning MSCs with caffeine has demonstrated reduced production of pro-inflammatory cytokines and improved disease status. Hypoxia and autophagy conditions have shown prospective application in increasing the immunomodulatory effects of MSCs. Preconditioning MSCs with pro-inflammatory cytokines, such as IFN-γ and IL-1β, has been found to enhance their immunosuppressive properties and increase the secretion of anti-inflammatory mediators. Combining IFN-γ with other pro-inflammatory cytokines further enhances the immunosuppressive effects of MSCs [156]. 

## 3. Concluding Remarks

Numerous studies, conducted mainly over the past decade, have provided encouraging evidence for the efficacy and safety of MSC-based therapies against RA. The therapeutic MSCs administered were derived from a variety of tissues and exhibited a wide spectrum of gene expression profiles, differentiation capacities, neurotrophin and cytokine release profiles, and extracellular vesicle contents. Both preclinical and clinical studies have found comparatively LDA, reduced levels of proinflammatory markers, and normalized T_reg_/T_h_17 and M1/M2 ratios following injection of MSCs or MSC-derived EVs, supporting the critical contribution of immune system suppression in these antirheumatic effects. Moreover, these anti-inflammatory effects have been shown to modulate osteoblastogenesis and osteoclastogenesis, thereby potentially improving joint tissue regeneration. Some human trials have also suggested improved QOL based on a variety of disease monitoring tools.

However, the therapeutic efficacies of MSCs and MSC-EVs vary according to tissue origin, harvesting methods, and EV extraction methods, among other factors, resulting in inconsistencies across studies [171,172,173]. Further, experts in the field [166] strongly recommend that future MSC-based preclinical and clinical investigations consider these issues moving forward. Several limitations hamper the development of MSC-based therapies, including the advanced age and poor health status of some patient donors, which reduce the quantity and quality of adult stem cells. Moreover, deciphering the precise mechanisms of action is complicated by the interactions between the host tissue and both MSCs and EVs. There are also no comprehensive pharmacodynamic and pharmacokinetic models for clinical simulations. Finally, the standardization of reagents and procedures for maintaining cell consistency remains challenging, and cost-effective, large-scale, and feasible manufacturing practices need to be developed for routine clinical application.

## Figures and Tables

**Table 1:** Properties of MSCs derived from four common source tissues for the treatment of arthritic diseases.

Tissue of Origin	Potential Advantages	Limitations	References
Bone marrow (BM-MSCs)	− Multiple clinical trials confirm safety and efficacy. − Possess remarkable differentiation potential. − Immunomodulatory effects and low immunogenicity. − BM-MSC-EVs enhance bone and cartilage repair with efficacy similar to the parent cell.	− Therapeutic efficacy is heavily dependent on the health and age of the donor. − Harvesting challenges include limited yield and risk of infection. − Mechanisms underlying therapeutic efficacy for RA remain underexplored.	[47,48,49,50,51,52,53,54]
Umbilical cord (UC-MSCs)	− Possess superior self-renewal and multi-differentiation capacities compared to other MSCs. − Easily and painlessly harvested. − Three- to four-fold greater proliferation rate than other MSCs. − Secrete multiple growth factors. − Numerous preclinical and clinical studies demonstrate treatment potential for RA. − UC-MSC-EVs demonstrate clinical restoration of the T_h_17/Treg balance.	− Early morphological changes. − Faster loss of amplification ability. − Lower attachment efficiency.	[55,56,57,58,59,60,61,62]
Umbilical cord blood (UCB-MSCs)	− Abundant and easily accessible source that is ethically non-controversial. − Distinct differentiation capacities compared to other sources. − Low risk of transmission: have a low risk of transmitting infections and mutations after transplantation. − Have low immunogenic and tumorigenic properties. − Higher immune modulatory effects by both direct immune cell contact and secretion factors. − Demonstrate higher proliferation rates. − Easier “Off-the-shelf” availability.	− Distribution and safety. − Heterogeneity challenge. − Low isolation yield limitation. − Good Manufacturing Practice (GMP) compliance concerns.	[63,64,65,66]
Adipose tissue (AD-MSCs)	− Easy to access with simple procedures and abundant yields from multiple sites. − Strong immunosuppressive effects. − Derived EVs suppress T_h_ cell development. − Amenable to large-scale production for autologous cell-based and cell-free therapy. − AD-MSC-EVs demonstrate effective preclinical cartilage and bone regeneration.	− AD-MSCs extracted from different sites demonstrate distinct traits. − Increased expression of HLA-ABC and HLA-DR in an environment of high IFN-γ. − May be unsuitable for allogenic application.Requires donor-matching for increased clinical efficiency.	[50,58,67,68,69,70,71,72,73]
Synovial membrane (SM-MSCs)	− Can be obtained from various sites for specific traits, including cotyloid fossa and paralabral synovium. − Higher proliferative capacity, greater multilineage differentiation capacity, and low immunogenicity than many other MSCs. − Hyperexpression of collagen II, aggrecan, and sex-determining region Y-box transcription factor 9 confer higher chondrogenic potential than MSCs from other sources. − Anticipated wide utilization for cartilage repair and joint homeostasis treatments. − SM-MSCs and BM-MSCs have greater osteogenic and adipogenic potentials than other MSCs.	− Relatively low-density expansion in vitro compared to BM-MSCs.	[74,75,76,77,78,79,80]

**Table 2:** Effects of the inflammatory microenvironment on the immunomodulatory potential of MSCs.

Tissue Microenvironment	MSC Phenotype	Characteristics	Expressed Molecules
Low inflammatory condition (Low levels of TNF-α and IFN-γ)	Proinflammatory mesenchymal stem cells (MSC1)	− Generally quiescent. − Expresses low levels of the immunosuppressive factors IDO and nitric oxide (NO). − Mainly associated with the early phase of inflammation. − Migrate to sites with high levels of proinflammatory cytokines via signaling between CC chemokine receptors (CCR1, 3, 7, 9, and 10) and CXC chemokines (CXCR3, 4, 5, and 6). − Activated by toll-like receptors (TLRs) such as TLR2 and TLR4. − Naïve cells induced to the MSC1 phenotype by TLR4. − Promote macrophage polarization to the M1 phenotype. − Increase osteogenic differentiation. − Decrease chondrogenic and adipogenic differentiation. − Enhance physiological healing.	− TLRs (TLR2 and TLR4) for activation and induction of proinflammatory cytokines (such as IL-6 and IL-8). − Activated T cell-secreted chemokines and macrophage inflammatory proteins (MIPs, MIP-1α and MIP-1β). − Factors secreted by inactivated T cells such asRANTES or CCL5, as well as CXCL9, CXCL10, and CXCL12.
High inflammatory microenvironment (High levels of TNF-α and IFN-γ)	Anti-inflammatory mesenchymal stem cells (MSC2)	− Derived from MSC1 by TLR3 stimulation. − Express high levels of immunosuppressive factors. − Impact homeostasis due to chronic inflammation. − Inhibit T cell proliferation and promote T_reg_ production.	− IDO, HLA-G5, TGF-β, galectins, IL-10, HGF, PGE2, heme oxygenase, and IFN-γ.

## Data Availability

Not applicable.
